# Prospective Association Between Problematic Mobile Phone Use and Eating Disorder Symptoms and the Mediating Effect of Resilience in Chinese College Students: A 1-Year Longitudinal Study

**DOI:** 10.3389/fpubh.2022.857246

**Published:** 2022-04-27

**Authors:** Shaojie Li, Guanghui Cui, Yongtian Yin, Kaixuan Tang, Lei Chen, Xinyao Liu

**Affiliations:** ^1^Shandong University of Traditional Chinese Medicine, Jinan, China; ^2^Xiangya School of Public Health, Central South University, Changsha, China

**Keywords:** problematic mobile phone use, eating disorder symptoms, resilience, mediation, college students, longitudinal study

## Abstract

A previous cross-sectional study found that problematic mobile phone use (PMPU) was associated with students' eating disorder symptoms. However, since the cross-sectional study cannot infer the causality and the direction of effect, the longitudinal relationship between the two and the mechanism behind this relationship are unclear. Therefore, the present study explores the prospective association between PMPU and eating disorder symptoms and related mediation mechanisms using a 1-year longitudinal study of 1,181 college students (from December 2019 [T1] to December 2020 [T2]). Survey tools used include the Mobile Phone Addiction Tendency Scale, the 10-item Connor-Davidson resilience scale, and the 12 item Short Form of the Eating Disorder Examination Questionnaire. The longitudinal relationship between PMPU and eating disorder symptoms and the mediating effect of resilience was analyzed using a cross-lagged model. The results showed that PMPU (β = 0.086, *P* < 0.01) and resilience (β = −0.145, *P* < 0.01) at T1 predicted eating disorder symptoms at T2, but not vice versa. PMPU was bidirectionally associated with resilience, and the prediction effect of PMPU at T1 to resilience at T2 (β = −0.151, *P* < 0.001) was higher than the prediction effect of resilience at T1 to PMPU at T2 (β = −0.134, *P* < 0.001). The standardized indirect effect of PMPU at T1 on eating disorder symptoms at T2 *via* resilience was significant (β = 0.022, 95% *CI* = 0.010~0.040, *P* < 0.001). Therefore, PMPU and resilience were predictive for eating disorder symptoms in college students, and resilience may play a mediating role in the prospective association between PMPU and eating disorder symptoms. This study provides new ideas and higher-level evidence for the development of prevention and intervention measures for college students' eating disorder symptoms.

## Introduction

Eating disorders are a class of psychiatric disorders characterized by abnormal diet or weight control behaviors, such as excessive dietary restrictions, body image disturbances (aversion to one's body shape or weight, excessive fear of gaining weight), or overeating (1). Previous studies have shown that eating disorders are associated with multiple adverse health outcomes such as type 2 diabetes (2), muscle dysmorphia (3), poor oral health (4), gastrointestinal disorders, malnutrition (5), and anxiety and depression symptoms (6). In addition, studies have found that eating disorders are associated with poor psychosocial functioning, such as alexithymia (7), increased risk of dropout (8), and illicit drug use (9). In recent years, with the increase in global overweight and obesity rates, eating disorder symptoms in non-clinical populations have received increasing attention from scholars. A meta-analysis showed the pooled lifetime and 12-month prevalence of eating disorder symptoms were 0.91% (95% CI, 0.48–1.71) and 0.43% (95% CI, 0.18–0.78), respectively (10). The prevalence of eating disorder symptoms in different age groups may be different. Previous studies have shown that eating disorder symptoms are the most common disorder in adolescence (11), and their prevalence peaks in late adolescence (12, 13), that is, early adulthood. A 14-year longitudinal study in the United States of 745 women aged 11 years old showed that the rate of their eating disorder symptoms increased year by year beginning at the age of 11 and showed significant stability after the age of 18 (14). During this subsequent period of stability, this demographic enters university, during which time they may experience high rates of eating disorder symptoms. A survey of 3,148 college students in six Asian countries showed that 11.5% of students are at risk of developing an eating disorder (15). Furthermore, research has also found that college students with eating disorder symptoms have higher suicidal tendencies (16), more depression and anxiety symptoms (17), and poorer quality of life (18). Considering these potential adverse effects and the higher risk of disease among college students, it is important to pay attention to the factors related to the occurrence of eating disorder symptoms in this group, as this is essential to designing related prevention and intervention measures to reduce their incidence.

Sociocultural models of eating disorders stated that the prevalence of body image concerns may exacerbate individual dissatisfaction or concerns about body image, lead to dietary restriction or overeating, and increase the risk of eating disorders (19). The rapid development of the Internet has widely disseminated the culture of dieting and bodybuilding (20, 21), which may create potential risks for eating disorders. Previous systematic reviews and meta-analysis studies also found that Internet use may be associated with eating disorder symptoms in college students (22, 23). Mobile phones provide the most convenient way for individuals to access the Internet anytime and anywhere (24). Due to the convenience and accessibility of mobile phones, they have become an indispensable communication tool for Chinese college students (25). Meanwhile, many problem behaviors, such as overuse and dependence, have also arisen through mobile phone use. Currently, academics conceptualize these problem behaviors as problematic mobile phone use (PMPU), which is also known as mobile phone addiction, smartphone addiction, or smartphone dependency, and refers to the uncontrolled or excessive use of smartphones that has a negative impact on daily life (26). Problematic mobile phone use was relatively common among Chinese college students. A meta-analysis showed that the combined prevalence of PMPU among Chinese college students was 23% (27). A large number of studies has found that PMPU may be related to many health problems of college students, such as heart palpitations, nausea (28), anxiety, and depression (29). In addition, studies also found PMPU may increase body image dissatisfaction (30), and produce unhealthy eating behaviors (31). However, few studies have focused on the relationship between PMPU and eating disorder symptoms. To our knowledge, only one cross-sectional study has explored the relationship between PMPU and eating disorder symptoms in college students (24). However, we cannot determine the direction of the association between PMPU and eating disorder symptoms because cross-sectional studies are unable to infer the limitations of causality. Therefore, it is necessary to conduct longitudinal research to further explore the prospective association between the two factors.

When exploring the relationship between PMPU and eating disorder symptoms, understanding the mechanism of this association is of great significance for the prevention of eating disorders in the future. Previous studies found that resilience can mediate the relationship between PMPU and poor health status (such as depression and anger) (32, 33). However, it was unclear whether resilience is able to mediate the link between PMPU and eating disorder symptoms. Before exploring this mechanism, it is necessary to understand the association of resilience with PMPU and eating disorders. Resilience refers to an individual's ability to actively cope with and adapt to life events or adversities (34). Resilience is often considered a positive psychological resource that helps explain the psychological mechanisms underlying life events and adverse health outcomes in individuals. For example, a previous study found resilience could neutralize the association between online risk exposure and negative psychological problems (35). Meanwhile, resilience is seen as a dynamic process and is influenced by many environmental and behavioral factors (36). As an unhealthy behavior, PMPU may deplete an individual's internal psychological resources, resulting in the individual's maladaptation to stress and difficulties. Studies have found that resilience is significantly negatively correlated with PMPU (37, 38). This suggests that PMPU may be an important predictor of resilience, but there is a lack of evidence from longitudinal studies. In addition, resilience plays an important role in the development of eating disorders. Early research has examined the role of resilience during the eating disorder recovery process (39). A previous longitudinal study of patients diagnosed with eating disorder symptoms found that resilience predicts that eating disorder symptoms will decrease over time (40). However, the studies discussed above were conducted in patients with diagnosed eating disorder symptoms, and it is not clear whether resilience can predict eating disorder symptoms in the non-clinical population. Obviously, exploring the relationship between resilience and eating disorder symptoms in non-clinical populations has important public health significance for early prevention and intervention. According to the test principle of the mediating effect, the mediating effect is established when both the independent variable and the mediating variable can predict the dependent variable and the independent variable can predict the mediating variable (41). Based on the above literature, it can be found that PMPU, resilience, and eating disorders are all pairwise correlated. Therefore, this study hypothesized that resilience may mediate the association between PMPU and eating disorder symptoms among college students.

In summary, the objectives of this study are as follows: (1) to explore the longitudinal association between PMPU, resilience, and eating disorder symptoms; and (2) to examine the mediating effect of resilience in the association between PMPU and eating disorder symptoms among college students.

## Materials and Methods

### Participants

We conducted a 1-year longitudinal study at a comprehensive university in Shandong Province, China. Before the study, we estimated the required sample size using the formula for epidemiological estimation of continuous outcomes: n = $n=\frac{{2\left(Z_{1-\alpha /2}+Z_{1-\beta }\right)}^{2}}{\delta^{2}}\;$ (42). In this study, we set the effect size (δ) for eating disorder symptoms at 0.20, the α at 0.05, and the power (1 – β) at 80%. Based on the parameters assumed above, *Z*_1−α/2_ was 1.96, *Z*_1−β_ was 0.84, and the calculated minimum sample size was 392. The stratified cluster sampling method was used to select 10 classes from freshman, sophomore, and junior levels, respectively, all of whom were 18 years of age or older. College students with clinically diagnosed diseases were excluded from the investigation. In December 2019 (T1), 1,235 participants completed the first survey. After 12 months (T2), 54 participants were not in school (due to leave and other reasons) and did not participate in the follow-up survey. In the end, 1,181 participants (599 women; age: mean = 18.91 years, SD = 0.85) participated in the two surveys (retention rate of 95.6%). Each survey takes ~30 min to complete, and each participant completed all survey content independently. This study was approved by the Medical Ethics Committee of the Second Affiliated Hospital of Shandong University of Traditional Chinese Medicine. All participants signed an informed consent document (on paper) before completing the survey.

### Measurements

#### PMPU (Both Waves)

PMPU was assessed using the Mobile Phone Addiction Tendency Scale (MPATS) (43), which is widely used in China (44). Participants rated the 16 items on a five-point Likert scale (from 1 “very inconsistent” to 5 “very consistent”). The total score, ranging from 16 to 80, is calculated by adding the scores of the 16 items. The higher the total score, the more serious the individual's PMPU. The Cronbach's alpha of this scale was 0.906 at T1 and 0.937 at T2.

#### Resilience (Both Waves)

The 10-item Connor-Davidson resilience scale (CD-RISC-10) (45) was used to measure the individuals' resilience. The scale has a total of 10 items and is scored using a four-point Likert scale (from 0 “never” to 4 “almost always”). The total score is obtained by adding the 10 items. A higher score indicates that the individual is more resilient. In this study, we used a validated Chinese version of the scale (46). The Cronbach's alpha of this scale was 0.925 at T1 and 0.947 at T2.

#### Eating Disorder Symptoms (Both Waves)

The 12 item Short Form of the Eating Disorder Examination Questionnaire was used to measure the individuals' eating disorder symptom severity (47). The scale has a single factor structure, with 12 items in total. Each item is scored using a four-point scale (from 0 “0 days” to 3 “6–7days”). A higher total score indicates that the individuals' eating disorder symptoms are more severe. In this study, we used a validated Chinese version of the scale (48). The Cronbach's alpha of this scale was 0.809 at T1 and 0.903 at T2.

#### Sociodemographic Variable

At T1, a self-designed sociodemographic information questionnaire was used to collect participants' age, sex, residence (urban or rural), self-reported height and weight [used to calculate body mass index (BMI)], self-rated health status (good, medium, or poor), and self-rated family economic level (high, medium, or low). Self-reported height and weight were again collected at T2.

### Statistical Methods

Data was analyzed using SPSS version 25.0 (IBM Corporation, Armonk NY, USA) and Amos version 23.0. Continuous variables, such as age and PMPU, were described by mean ± standard deviation (SD), while categorical variables, such as sex and residence, were described by *n* (%). Since all measurements were self-reported, we used Harman's single-factor test (49) to assess the presence of common method bias. According to the test criteria, when the variance explained by the first common factor is less than 50%, it means there is no serious common method bias (50). Spearman correlation analysis was used to investigate the relationship between PMPU, resilience, and eating disorder symptoms. The structural equation model with maximum likelihood estimation approach was used to construct a cross-lagged model to investigate the longitudinal relationship between PMPU, resilience, and eating disorder symptoms based on the control of sex, age, residence, BMI, self-rated health status, and self-rated family economic level at T1. Chi-square/degree of freedom, the comparative fit index (CFI), the Tucker–Lewis index (TLI), the root–mean–square error of approximation (RMSEA), and the standard root–mean–square (SRMR) were used to evaluate the model fit. When the chi-square/degree of freedom is <5, CFI and TLI are >0.90, and RMSEA and SRMR are <0.08, then the model fit is acceptable (51). According to Cole and Maxwell's recommendation regarding two waves of longitudinal studies (52), we calculated the product of the regression coefficient from T1 PMPU to T2 resilience and the regression coefficient from T1 resilience to T2 eating disorder symptoms, and we used the bootstrap method for the significance test. When the 95% confidence interval (CI) does not contain 0, then the mediating effect of resilience is statistically significant.

## Results

### Descriptive Statistics

The data on the characteristics of sociodemographic variables, PMPU, resilience, and eating disorder symptoms are shown in Table 1.

**Table 1:** Descriptive statistic for all variables (*N* = 1,181).

**Variables**	***n*** **(%)/M ±SD**	**Range**
Age	18.91 ± 0.85	18-22
**Sex**
Male	582 (49.3)	
Female	599 (50.7)	
**Residence**
Urban	518 (43.9)	
Rural	663 (56.1)	
**Self-rated health status**
Good	580 (49.1)	
Medium	418 (35.4)	
Poor	183 (15.5)	
**Self-rated family economic level**
High	190 (16.1)	
Medium	827 (70.0)	
Low	164 (13.9)	
BMI-T1	20.81 ± 2.70	14.20-32.39
BMI-T2	20.98 ± 3.11	14.35-35.56
PMPU-T1	39.20 ± 12.27	16.00-80.00
Resilience-T1	26.74 ± 6.92	0.00-40.00
Eating disorders-T1	6.49 ± 4.95	0.00-26.00
PMPU-T2	37.08 ± 13.62	16.00-77.00
Resilience-T2	27.36 ± 7.61	0.00-40.00
Eating disorders-T2	6.39 ± 6.80	0.00-30.00

*M, mean; SD, standard deviation*.

### Common Method Bias Test

The results showed that the characteristic values of a total of 14 factors were >1, and that the first common factor explained 23.5% of the total variation, which is <50%. This indicates there was no serious common method bias in this study.

### Correlation Analyses

Table 2 shows the correlations for BMI, PMPU, resilience, and eating disorder symptoms at two time points. The results show significant positive correlations between BMI, PMPU, resilience, and eating disorder symptoms of both T1 and T2, suggesting that the four factors have shown stability across time within a year. In addition, resilience at T1 and T2 was significantly negatively correlated with PMPU and eating disorder symptoms, and PMPU was significantly positively correlated with eating disorder symptoms at two time points, indicating there was a certain synchronous correlation between the three factors at different time points. Moreover, BMI was positively associated with eating disorder symptoms at two time points.

**Table 2:** Spearman correlations analyses for BMI, PMPU, resilience, and eating disorders at two time points.

**Variables**	**1**	**2**	**3**	**4**	**5**	**6**	**7**	**8**
1.BMI-T1	1.000							
2.BMI-T2	0.624**	1.000						
3.PMPU-T1	0.002	0.011	1.000					
4.PMPU-T2	0.037	0.040	0.527**	1.000				
5.Resilience-T1	−0.045	−0.017	−0.407**	−0.347**	1.000			
6.Resilience-T2	−0.012	−0.009	−0.319**	−0.480**	0.484**	1.000		
7.Eating disorders-T1	0.280**	0.207**	0.360**	0.240**	−0.197**	−0.190**	1.000	
8.Eating disorders-T2	0.237**	0.269**	0.276**	0.325**	−0.257**	−0.342**	0.456**	1.000

** *P < 0.01*.

### Cross-Lagged Model and Mediation Analyses

**Figure 1:**
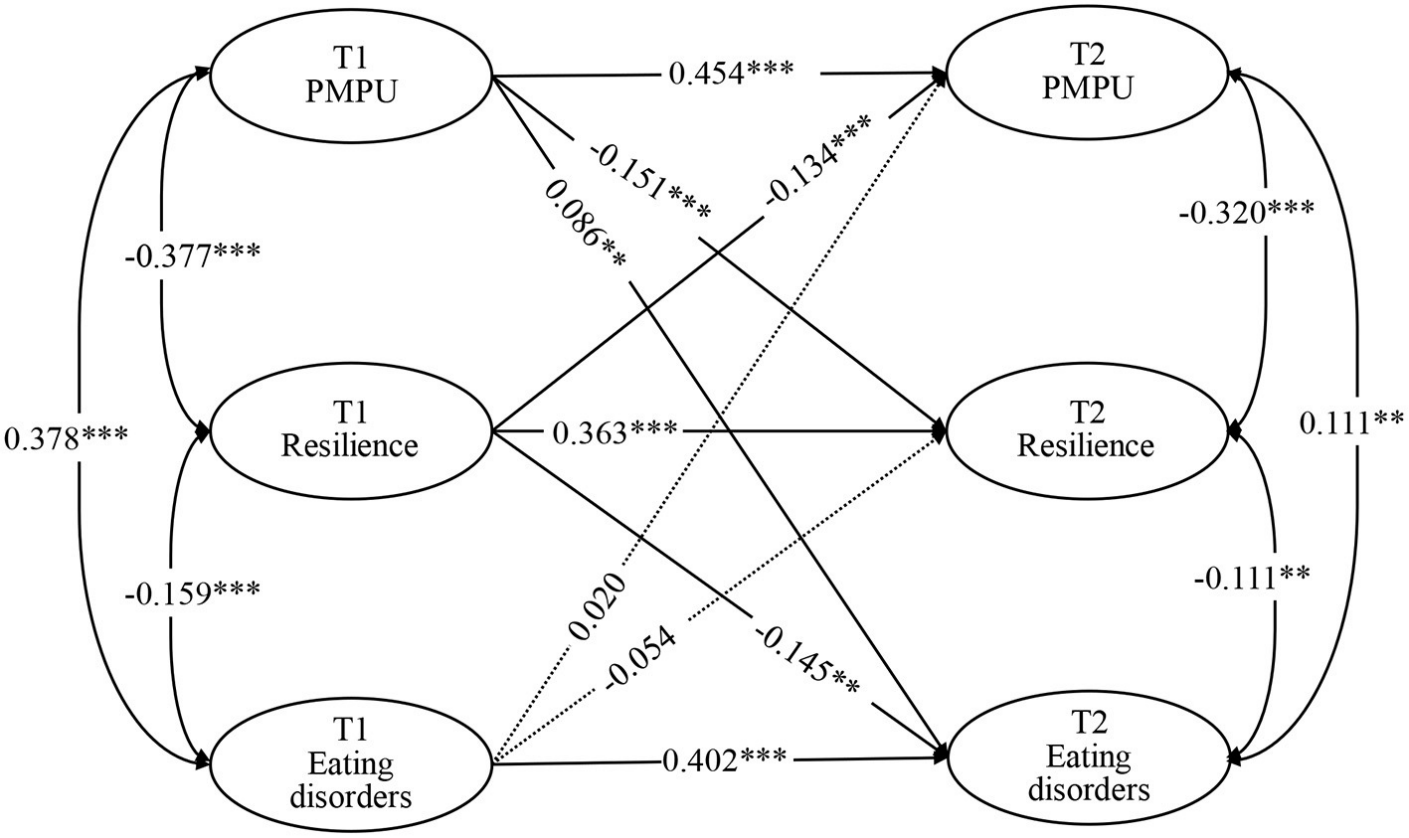
Cross-lagged model for PMPU, resilience, and eating disorders at two time points. ^**^P < 0.01, ^***^P < 0.001.

The results showed that the cross-lagged model has a fit index (χ^2^/*df* = 3.036, CFI = 0.989, TLI = 0.943, RMSEA = 0.042, SRMR = 0.024). The cross-lagged model showed that PMPU (β = 0.086, *P* < 0.01) and resilience (β = −0.145, *P* < 0.01) at T1 predicted eating disorder symptoms at T2, but not vice versa. PMPU was bidirectionally associated with resilience, and the prediction effect of PMPU at T1 to resilience at T2 (β = −0.151, *P* < 0.001) was higher than the prediction effect of resilience at T1 to PMPU at T2 (β = −0.134, *P* < 0.001). More details are displayed in Figure 1; Table 3.

**Table 3:** Bootstrapped estimation of each path of the reciprocal cross-lagged model.

**Path**	**Effect**	**SE**	**LLCI**	**ULCI**	***P*** **-value**
PMPU-T1 to PMPU-T2	0.454	0.031	0.391	0.512	<0.001
Resilience-T1 to resilience-T2	0.363	0.031	0.302	0.422	<0.001
Eating disorder symptoms-T1 to eating disorder symptoms-T2	0.402	0.030	0.344	0.459	<0.001
PMPU-T1 to resilience-T2	−0.151	0.027	−0.204	−0.098	<0.001
PMPU-T1 to eating disorder symptoms -T2	0.086	0.031	0.024	0.147	0.008
Resilience-T1 to PMPU-T2	−0.134	0.028	−0.187	−0.078	<0.001
Resilience T1 to eating disorder symptoms-T2	−0.145	0.041	−0.229	−0.066	0.001
Eating disorder symptoms-T1 to PMPU-T2	0.020	0.028	−0.035	0.075	0.462
Eating disorder symptoms-T1 to resilience-T2	−0.054	0.030	−0.114	0.003	0.059

*LLCI, lower limit confidence interval 95%; ULCI, upper limit confidence interval 95%*.

In addition, the standardized indirect effect of PMPU at T1 on eating disorder symptoms at T2 *via* resilience was significant (β = 0.022, 95% CI = 0.010~0.040, *P* < 0.001); that is, resilience mediated the relationship between PMPU and eating disorder symptoms.

## Discussion

Our study clarifies the prospective association between PMPU, resilience, and eating disorder symptoms in college students using a 1-year follow-up survey. Our results show that early PMPU and resilience predict follow-up eating disorder symptoms, and PMPU can indirectly affect eating disorder symptoms through resilience. These findings provide scientific evidence for the early prevention of and intervention against eating disorder symptoms.

This study found that PMPU at T1 predicted eating disorder symptoms at T2, but not vice versa. Although previous cross-sectional studies have found that PMPU is associated with eating disorder symptoms (24), it is impossible to determine whether PMPU causes eating disorder symptoms or eating disorder symptoms cause PMPU. Our findings further clarified that PMPU is an antecedent variable for eating disorder symptoms, rather than the reverse. One possible reason to explain this result is that individuals with PMPU are more susceptible to sociocultural influences. At present, Chinese college students' smartphones were mainly used for accessing web pages, socializing, and taking selfies (53). Previous studies found that news about beauty and slimness prevalent on the web may cause frequent users to be dissatisfied with their body image and appearance (54, 55). In addition, studies also found that adolescents who frequently take selfies and post selfies on social media are more likely to develop eating disorder symptoms, as social media depictions and dissemination of the idealized female body can exacerbate habitual monitoring of physical appearance and body anxiety (56, 57). The above literature provides indirect support for the results of this study; that is, PMPU may lead individuals to pay more attention to body image, thereby causing them to reduce their food intake or deliberately stop eating, resulting in eating disorder symptoms. Moreover, the displacement effect assumption holds that the more time individuals watch TV, the less time and energy they spend on other activities (58), and this also applies to smartphones. This means that because individuals with PMPU use their smartphones for too long, they may reduce their eating time and choose to eat fast food or not eat at all, which increases their risk of developing eating disorder symptoms. In addition, individuals with PMPU are prone to sedentary behavior (59), which increases the likelihood of becoming overweight or obese (60) to a certain extent. Previous studies have found that individuals with a higher BMI are vulnerable to discrimination, which can cause dissatisfaction with weight and induce eating disorder symptoms (61). Furthermore, research has also found that exposure to screen media can cause children and adolescents to increase their food intake while watching (62), and similar phenomena may also occur during smartphone use. Given the limited literature on the association of PMPU with eating disorders, our further interpretation of the association is also limited. It is necessary to conduct further observational studies in more countries and regions in the future to better understand the relationship between the two factors.

The results of the cross-lagged model show that resilience at T1 negatively predicted eating disorder symptoms at T2; that is, the higher one's resilience, the lower the risk of developing an eating disorder. However, the association between eating disorder symptoms at T1 and resilience at T2 was not significant. This result infers resilience from the patients with eating disorder symptoms to the college student population, extending the findings of previous studies. Many studies have confirmed the positive effect of resilience on individual health status (63, 64). For example, resilience is significantly positively correlated with the quality of life of adolescents (65) and negatively correlated with negative emotions, such as depression (66) and anxiety (67). In terms of eating-related aspects, research has also found that the higher the resilience of college students, the fewer the symptoms of food addiction and eating disorders they exhibit (68, 69). These studies further support that resilience has a positive effect on the reduction of the rate of eating disorder symptoms. In addition, the resilience framework proposed by Kumpfer (70) explains that resilience can help individuals actively cope with and adapt to the impact of negative factors after encountering negative life experiences. The epigenetic model of the etiology of eating disorder symptoms indicates that life stress is an important cause of eating disorder symptoms (71). Obviously, high resilience can help individuals actively adapt to stressful events, which helps reduce the incidence of bad eating behaviors. Meanwhile, individuals with lower resilience may respond negatively to stress and traumatic events, and may adopt disordered eating, overeating, and other poor eating behaviors to relieve stress, which greatly increases the risk of developing an eating disorder.

Another important finding of this study is that resilience can mediate the association between PMPU and eating disorder symptoms. This result deepens our understanding of the underlying mechanisms of PMPU and eating disorder symptoms. Previous studies have also identified a potential mediating effect of resilience in the development of eating disorder symptoms; that is resilience could mediate the relationship between posttraumatic stress disorder symptoms and disordered eating in college women (69). In this study, the mediation mechanism can be divided into two stages: (1) PMPU-reduced resilience, and (2) lower resilience-induced eating disorder symptoms. The latter has already been explained above. For the former, some studies have focused on the relationship between PMPU and resilience (72), but as we mentioned earlier, there is also a lack of evidence from longitudinal studies. Our research indicates that PMPU and resilience are bidirectionally related, and early PMPU has a greater predictive effect on follow-up resilience. Considering that the prediction effect of PMPU at T1 to resilience at T2 was higher than the prediction effect of resilience at T1 to PMPU at T2, it can also be said that PMPU is an antecedent variable of resilience. Research has shown that resilience is an important internal resource formed in the development process of young people, and such resources can promote and protect healthy growth (73). However, research has also found that PMPU can reduce self-control (74), thereby consuming individual internal resources and reducing resilience. The possible explanation for the mediating effect of resilience is that PMPU may increase individual body image stress, consumes positive psychological resources, and reduces resilience to cope with stress, while low resilience makes individuals more likely to adopt poor eating behaviors, resulting in eating disorder symptoms. Therefore, if college students with PMPU can accept early psychological intervention programs to improve their resilience, such programs may, to a certain extent, be able to reduce their likelihood of developing eating disorder symptoms caused by PMPU.

According to our research, it may be a good idea to develop prevention and intervention programs related to PMPU and eating disorder symptoms in college students from the perspective of resilience. Previous randomized controlled trials conducted among American college students have found that interventions related to resilience significantly improved participants' mental health (75). A meta-analysis also showed that school-based resilience interventions can effectively reduce illicit substance use in adolescents (76). These studies suggest that interventions related to resilience may also have a positive impact on PMPU and eating disorder symptoms, but as far as we know, no relevant intervention studies currently exist. However, a meta-analysis also found that existing resilience intervention research has problems, such as the small intervention effect, small sample sizes, and poor compliance, and has made relevant recommendations for the development of high-quality intervention research (77). Therefore, we suggest that future research should learn from the problems and suggestions of previous studies, conduct resilience intervention studies on PMPU and eating disorder symptoms in college students, properly deal with all possible biases, and further understand whether resilience intervention can help reduce PMPU and eating disorder symptoms to provide a higher level of evidence for future prevention and intervention measures.

This study has the following advantages. First, as far as we know, this is the first study to investigate the longitudinal association between PMPU, resilience, and eating disorder symptoms, which provides a higher level of research evidence for understanding the direction of the association between the three. Second, we explored the mechanism of association between PMPU and eating disorder symptoms for the first time and found that resilience played a mediating role between the two, which provided a new direction for the prevention of eating disorder symptoms in college students. Third, all surveys were conducted using scales that have been tested by psychometrics, and the quality of the data is credible and reliable.

This study has the following limitations. First, we only conducted two waves of investigations. Although previous studies have suggested that two waves of research can also be used to test the mediation effect (78), since there is no continuous time relationship between PMPU, resilience, and eating disorder symptoms in the two waves of the present study, the mediating effect of resilience has a certain limitation. In the future, three waves of longitudinal study will be needed to test whether the mediating role of resilience can be replicated. Second, due to the short 1-year follow-up period, it remains unclear whether the longitudinal relationship of the three factors in this study will change over time. Long-term follow-up research is needed to further determine the direction of associations among these factors. Third, the two surveys were conducted in the form of self-reports, and the results may be subject to recall and information bias. Finally, this study only selected college students from one university as the research object, which may introduce selection bias. In the future, a multi-center research design will be needed to verify whether the results of this study are applicable to college students in other regions of China.

## Conclusions

This study found that PMPU and resilience are predictive for eating disorder symptoms in college students. Resilience may play a mediating role in the prospective association between PMPU and eating disorder symptoms.

## Data Availability Statement

The datasets can be made available to any interested person(s) contacting the corresponding author via email.

## Ethics Statement

The studies involving human participants were reviewed and approved by Medical Ethics Committee of the Second Affiliated Hospital of Shandong University of Traditional Chinese Medicine. The patients/participants provided their written informed consent to participate in this study.

## Author Contributions

SL participated in questionnaire design, data collection and statistical analysis, and wrote the first draft of the manuscript. GC conducted data collection and gave comments on the draft. YY revised the first draft. KT, LC, and XL participated in data collection. All authors approved submission of the final manuscript.

## Funding

This study was supported by the Social Science Funding of Shandong Province (21CJYJ29). The source of funding had no role in study design, data collection, analysis, interpretation, or manuscript writing.

## Conflict of Interest

The authors declare that the research was conducted in the absence of any commercial or financial relationships that could be construed as a potential conflict of interest.

## Publisher's Note

All claims expressed in this article are solely those of the authors and do not necessarily represent those of their affiliated organizations, or those of the publisher, the editors and the reviewers. Any product that may be evaluated in this article, or claim that may be made by its manufacturer, is not guaranteed or endorsed by the publisher.
